# Pneumatic Plugger

**Published:** 1883-08

**Authors:** 


### PNEUMATIC PLUGGER.

Dr. C. F. Rich, of Saratoga Springs, has devised a pneumatic plugger that is capable of excellent work. It consists of a very simple air-pump, which may be attached to a water or other motor, and placed beneath the floor, or in an adjoining room. A rubber pipe connects the pump with a hand-piece, in appearance not unlike an automatic plugger, in which is a diaphragm, and to this is attached the piston-like hammer. The rubber connecting pipe is led underneath or behind the chair, and is fitted with a cut-off, which, by simple pressure of the foot, controls the air supply and suspends the action of the plugger while picking up and putting in place the gold. It is intended that the connecting pipe shall be attached to the operating table, and at this point is a cock which may be turned so as entirely to stop the action when the foot is removed from the pedal cut-off. The plugger is adapted to either the Snow & Lewis, or the Salmon points.

We have been using it attached to a dental engine, which does not operate it to the best advantage, but even under these unfavorable circumstances are pleased with the work which it has done. The blow is direct, its force may be varied by the simple revolution of a milled band upon the part held between the fingers, while the impact is a very close imitation of that of the hand mallet. The rapidity of the blows depends upon the revolutions of the motor, but it is sufficient for the rapid condensation of gold. The use of this instrument, like that of the electric plugger, relieves the operator from very much of the nervous constraint attendant upon mere hand work, and results in the saving of valuable time.

Dr. Rich has also devised a very simple and convenient rack for the holding of the points to be used in the plugger.

